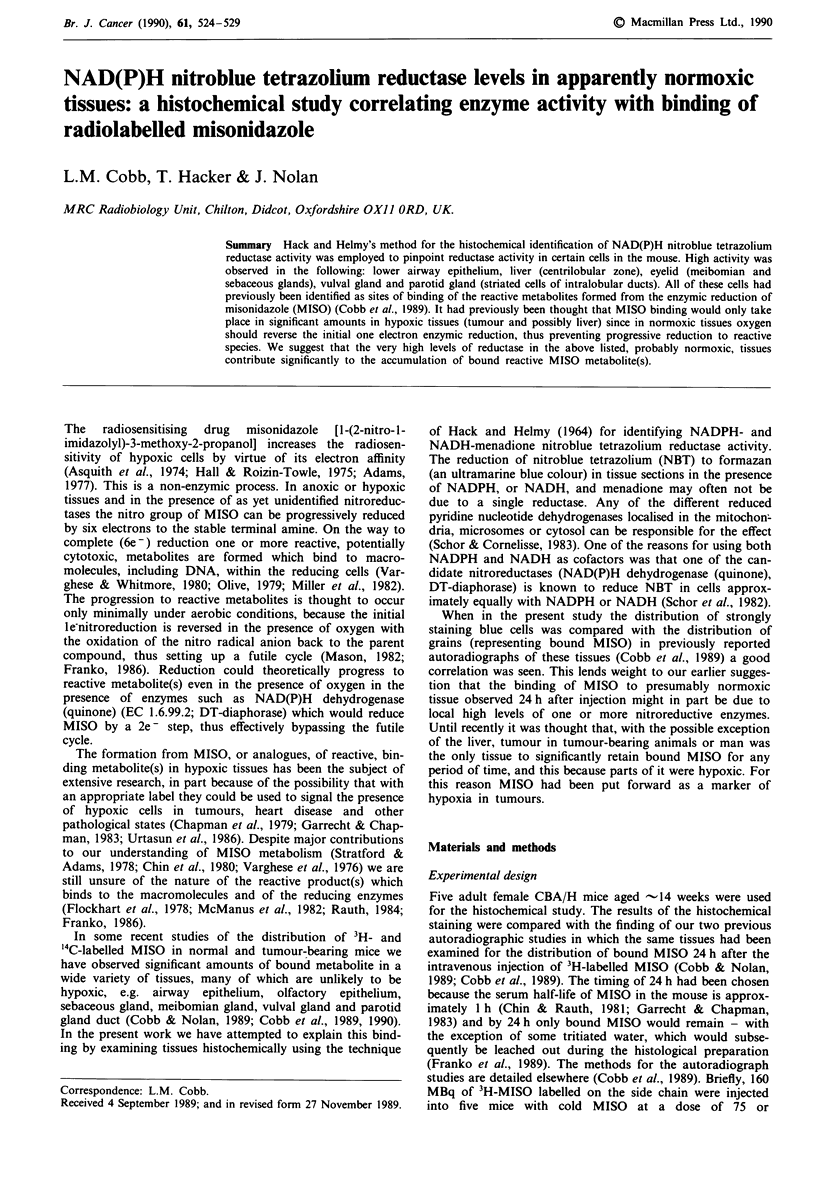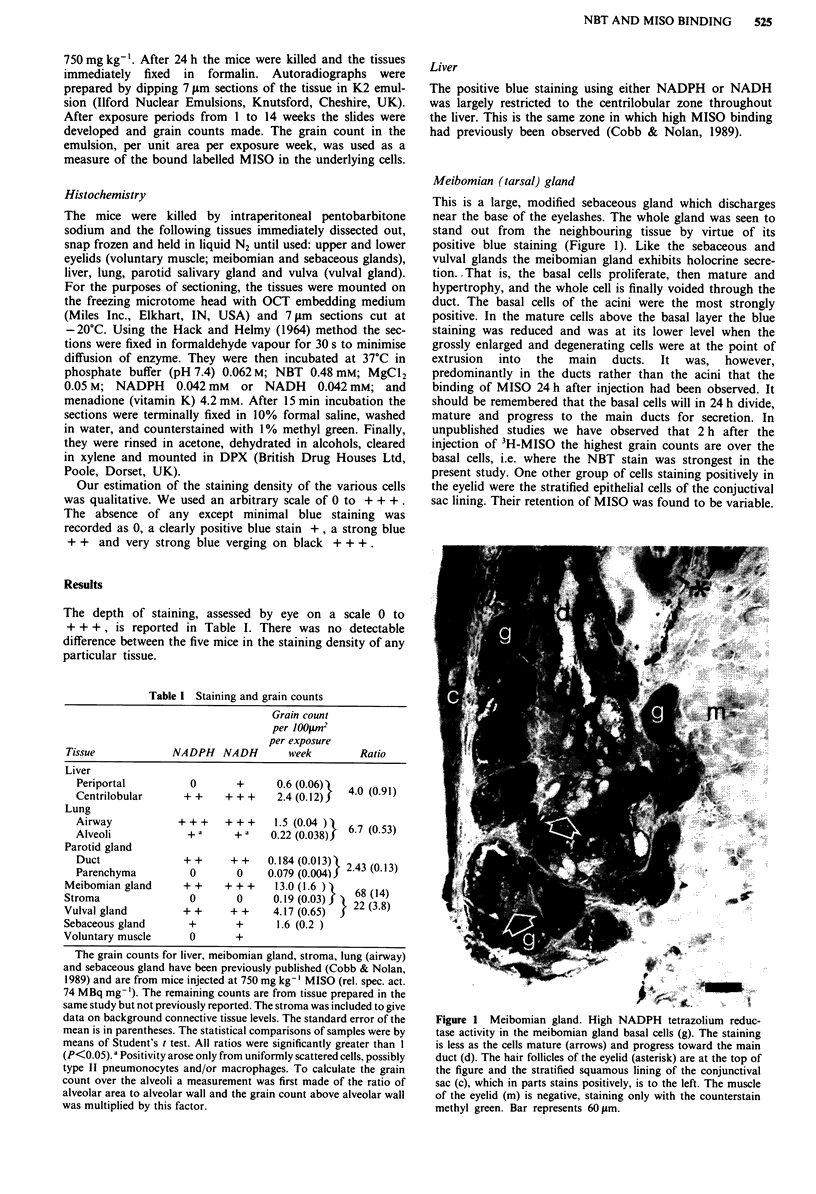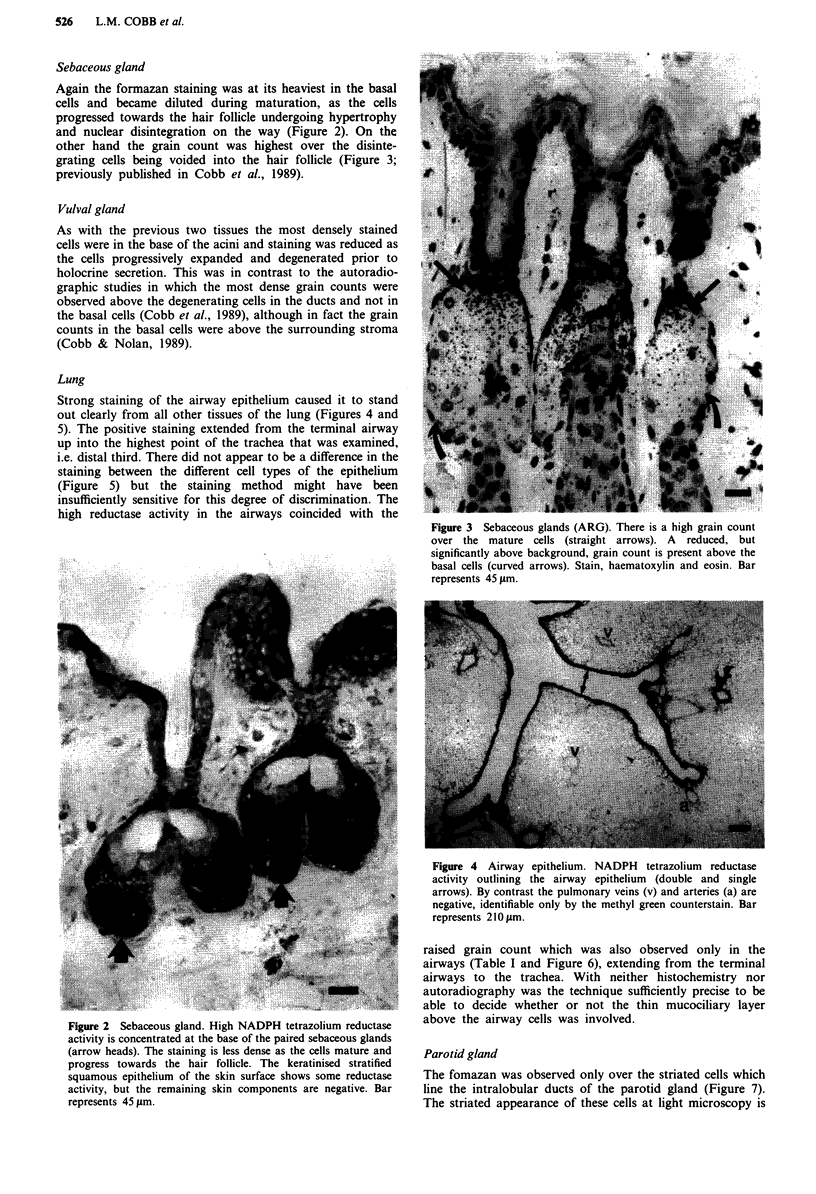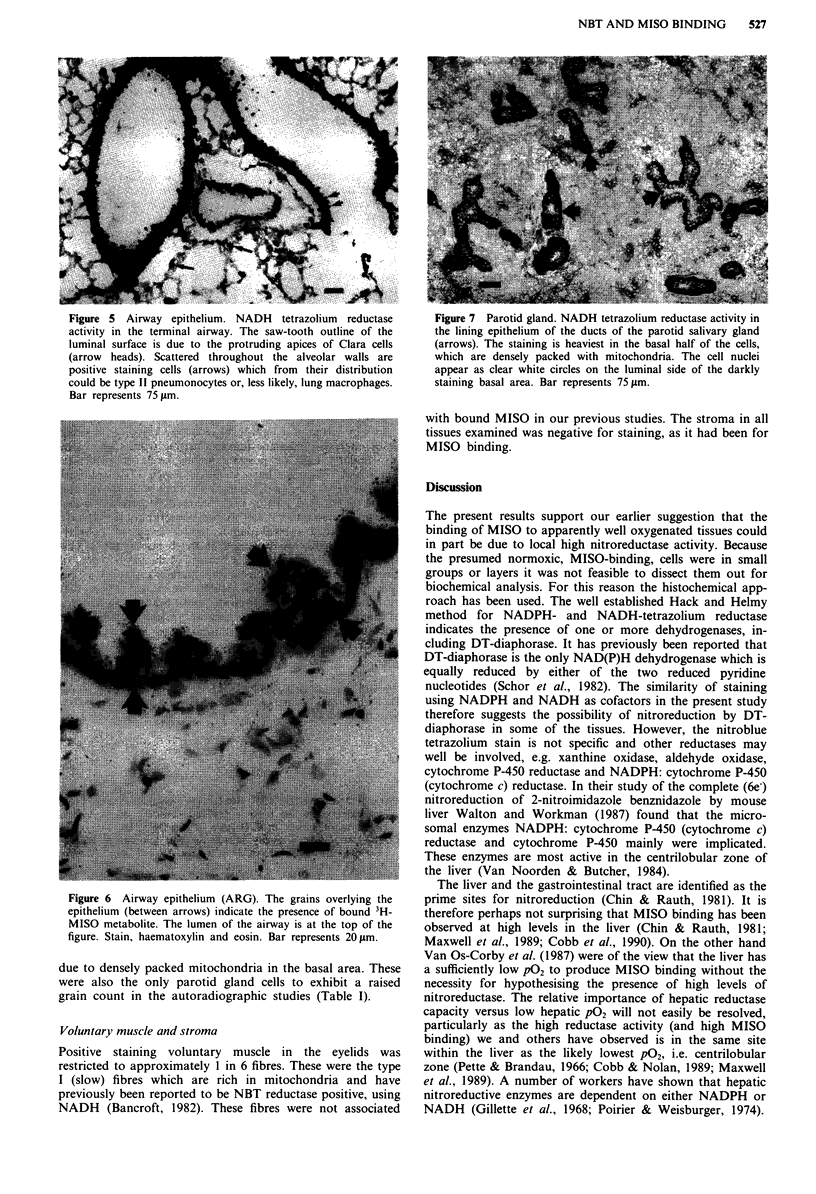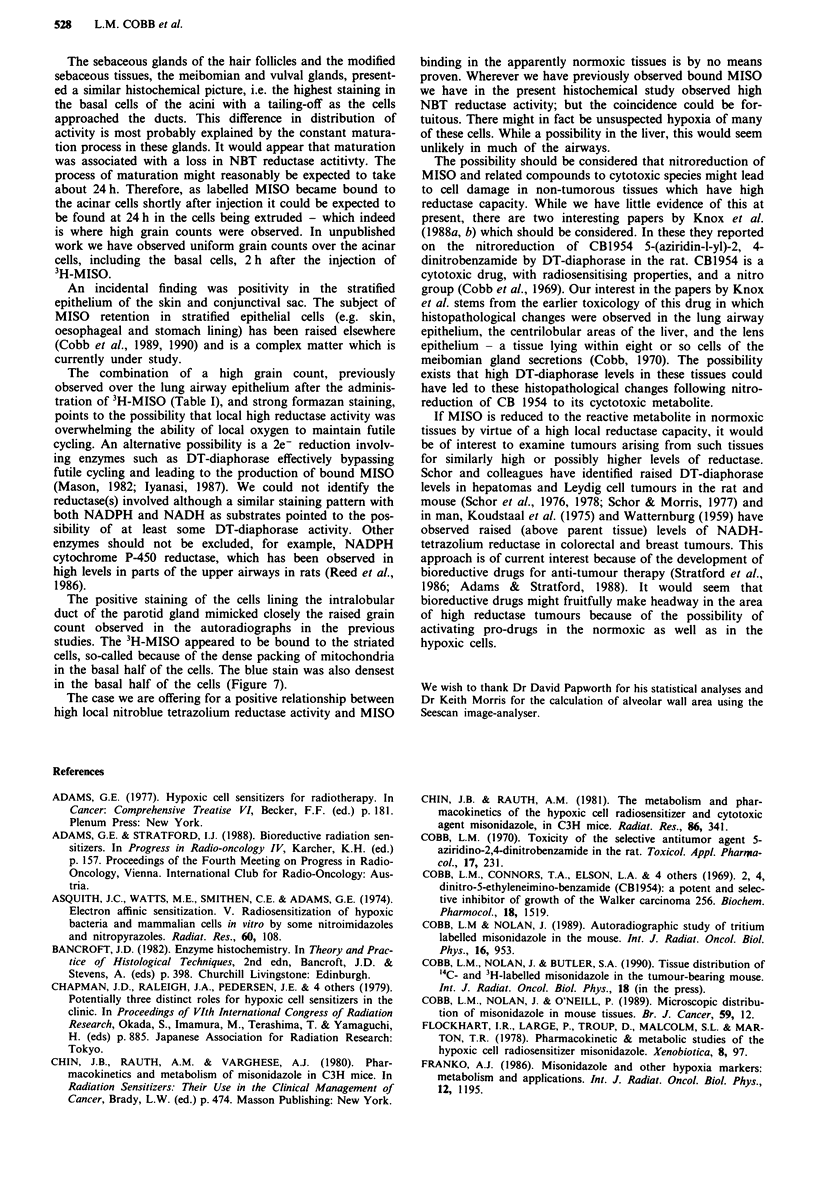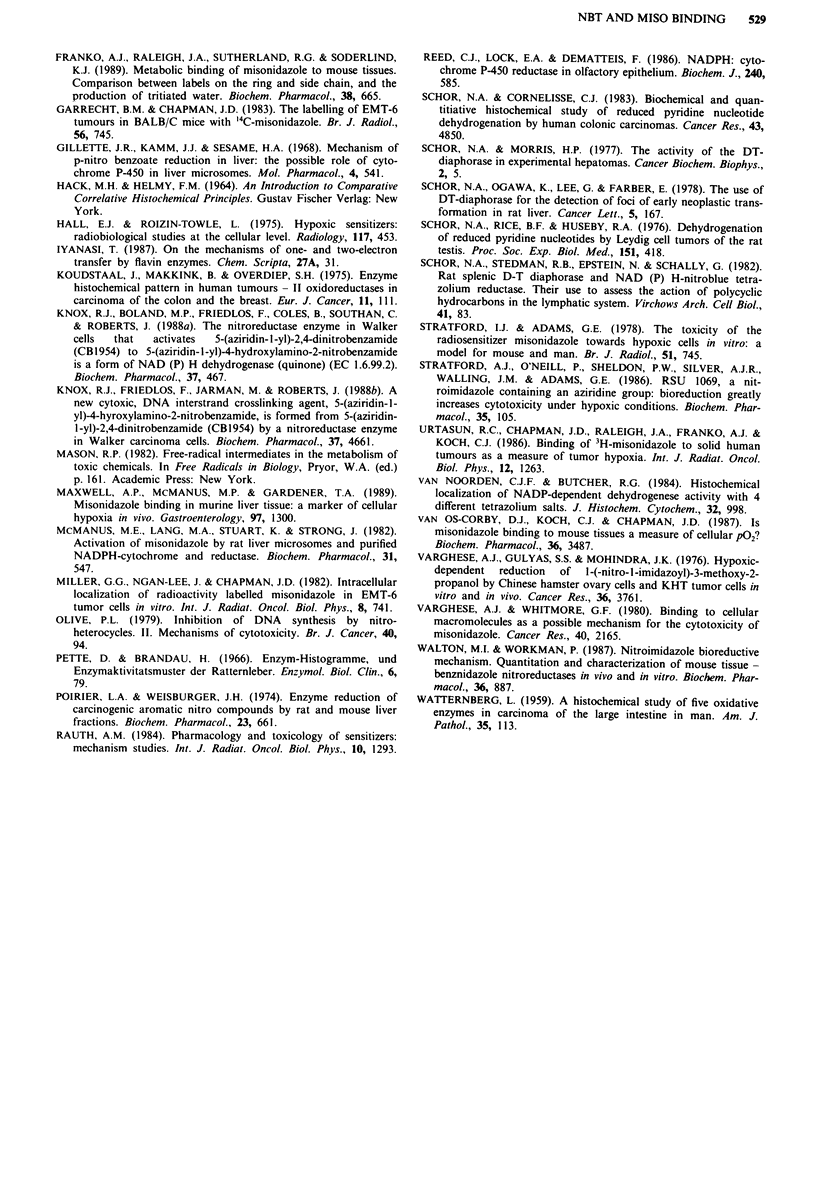# NAD(P)H nitroblue tetrazolium reductase levels in apparently normoxic tissues: a histochemical study correlating enzyme activity with binding of radiolabelled misonidazole.

**DOI:** 10.1038/bjc.1990.118

**Published:** 1990-04

**Authors:** L. M. Cobb, T. Hacker, J. Nolan

**Affiliations:** MRC Radiobiology Unit, Didcot, Oxfordshire, UK.

## Abstract

**Images:**


Br. J. Cancer (1990), 61, 524-529 © Macmillan Press Ltd., 1990

### NAD(P)H nitroblue tetrazolium reductase levels in apparently normoxic tissues: a histochemical study correlating enzyme activity with binding of radiolabelled misonidazole

L.M. Cobb, T. Hacker & J. Nolan

*MRC Radiobiology Unit, Chilton, Didcot, Oxfordshire OX11 0RD, UK.*

**Summary** Hack and Helmy's method for the histochemical identification of NAD(P)H nitroblue tetrazolium reductase activity was employed to pinpoint reductase activity in certain cells in the mouse. High activity was observed in the following: lower airway epithelium, liver (centrilobular zone), eyelid (meibomian and sebaceous glands), vulval gland and parotid gland (striated cells of intralobular ducts). All of these cells had previously been identified as sites of binding of the reactive metabolites formed from the enzymic reduction of misonidazole (MISO) (Cobb *et al.*, 1989). It had previously been thought that MISO binding would only take place in significant amounts in hypoxic tissues (tumour and possibly liver) since in normoxic tissues oxygen should reverse the initial one electron enzymic reduction, thus preventing progressive reduction to reactive species. We suggest that the very high levels of reductase in the above listed, probably normoxic, tissues contribute significantly to the accumulation of bound reactive MISO metabolite(s).

The radiosensitising drug misonidazole [1-(2-nitro-1-imidazolyl)-3-methoxy-2-propanol] increases the radiosensitivity of hypoxic cells by virtue of its electron affinity (Asquith *et al.*, 1974; Hall & Roizin-Towle, 1975; Adams, 1977). This is a non-enzymic process. In anoxic or hypoxic tissues and in the presence of as yet unidentified nitroreductases the nitro group of MISO can be progressively reduced by six electrons to the stable terminal amine. On the way to complete ($6e^-$) reduction one or more reactive, potentially cytotoxic, metabolites are formed which bind to macromolecules, including DNA, within the reducing cells (Varghese & Whitmore, 1980; Olive, 1979; Miller *et al.*, 1982). The progression to reactive metabolites is thought to occur only minimally under aerobic conditions, because the initial $1e^-$ nitroreduction is reversed in the presence of oxygen with the oxidation of the nitro radical anion back to the parent compound, thus setting up a futile cycle (Mason, 1982; Franko, 1986). Reduction could theoretically progress to reactive metabolite(s) even in the presence of oxygen in the presence of enzymes such as NAD(P)H dehydrogenase (quinone) (EC 1.6.99.2; DT-diaphorase) which would reduce MISO by a $2e^-$ step, thus effectively bypassing the futile cycle.

The formation from MISO, or analogues, of reactive, binding metabolite(s) in hypoxic tissues has been the subject of extensive research, in part because of the possibility that with an appropriate label they could be used to signal the presence of hypoxic cells in tumours, heart disease and other pathological states (Chapman *et al.*, 1979; Garrecht & Chapman, 1983; Urtasun *et al.*, 1986). Despite major contributions to our understanding of MISO metabolism (Stratford & Adams, 1978; Chin *et al.*, 1980; Varghese *et al.*, 1976) we are still unsure of the nature of the reactive product(s) which binds to the macromolecules and of the reducing enzymes (Flockhart *et al.*, 1978; McManus *et al.*, 1982; Rauth, 1984; Franko, 1986).

In some recent studies of the distribution of $^3H$- and $^{14}C$-labelled MISO in normal and tumour-bearing mice we have observed significant amounts of bound metabolite in a wide variety of tissues, many of which are unlikely to be hypoxic, e.g. airway epithelium, olfactory epithelium, sebaceous gland, meibomian gland, vulval gland and parotid gland duct (Cobb & Nolan, 1989; Cobb *et al.*, 1989, 1990). In the present work we have attempted to explain this binding by examining tissues histochemically using the technique of Hack and Helmy (1964) for identifying NADPH- and NADH-menadione nitroblue tetrazolium reductase activity. The reduction of nitroblue tetrazolium (NBT) to formazan (an ultramarine blue colour) in tissue sections in the presence of NADPH, or NADH, and menadione may often not be due to a single reductase. Any of the different reduced pyridine nucleotide dehydrogenases localised in the mitochondria, microsomes or cytosol can be responsible for the effect (Schor & Cornelisse, 1983). One of the reasons for using both NADPH and NADH as cofactors was that one of the candidate nitroreductases (NAD(P)H dehydrogenase (quinone), DT-diaphorase) is known to reduce NBT in cells approximately equally with NADPH or NADH (Schor *et al.*, 1982).

When in the present study the distribution of strongly staining blue cells was compared with the distribution of grains (representing bound MISO) in previously reported autoradiographs of these tissues (Cobb *et al.*, 1989) a good correlation was seen. This lends weight to our earlier suggestion that the binding of MISO to presumably normoxic tissue observed 24 h after injection might in part be due to local high levels of one or more nitroreductive enzymes. Until recently it was thought that, with the possible exception of the liver, tumour in tumour-bearing animals or man was the only tissue to significantly retain bound MISO for any period of time, and this because parts of it were hypoxic. For this reason MISO had been put forward as a marker of hypoxia in tumours.

### Materials and methods

### *Experimental design*

Five adult female CBA/H mice aged ~14 weeks were used for the histochemical study. The results of the histochemical staining were compared with the finding of our two previous autoradiographic studies in which the same tissues had been examined for the distribution of bound MISO 24 h after the intravenous injection of $^3H$-labelled MISO (Cobb & Nolan, 1989; Cobb *et al.*, 1989). The timing of 24 h had been chosen because the serum half-life of MISO in the mouse is approximately 1 h (Chin & Rauth, 1981; Garrecht & Chapman, 1983) and by 24 h only bound MISO would remain – with the exception of some tritiated water, which would subsequently be leached out during the histological preparation (Franko *et al.*, 1989). The methods for the autoradiograph studies are detailed elsewhere (Cobb *et al.*, 1989). Briefly, 160 MBq of $^3H$-MISO labelled on the side chain were injected into five mice with cold MISO at a dose of 75 or

Correspondence: L.M. Cobb.
Received 4 September 1989; and in revised form 27 November 1989.

750 mg $kg^{-1}$. After 24 h the mice were killed and the tissues immediately fixed in formalin. Autoradiographs were prepared by dipping 7 μm sections of the tissue in K2 emulsion (Ilford Nuclear Emulsions, Knutsford, Cheshire, UK). After exposure periods from 1 to 14 weeks the slides were developed and grain counts made. The grain count in the emulsion, per unit area per exposure week, was used as a measure of the bound labelled MISO in the underlying cells.

### *Histochemistry*

The mice were killed by intraperitoneal pentobarbitone sodium and the following tissues immediately dissected out, snap frozen and held in liquid $N_2$ until used: upper and lower eyelids (voluntary muscle; meibomian and sebaceous glands), liver, lung, parotid salivary gland and vulva (vulval gland). For the purposes of sectioning, the tissues were mounted on the freezing microtome head with OCT embedding medium (Miles Inc., Elkhart, IN, USA) and 7 μm sections cut at −20°C. Using the Hack and Helmy (1964) method the sections were fixed in formaldehyde vapour for 30 s to minimise diffusion of enzyme. They were then incubated at 37°C in phosphate buffer (pH 7.4) 0.062 M; NBT 0.48 mM; $MgCl_2$ 0.05 M; NADPH 0.042 mM or NADH 0.042 mM; and menadione (vitamin K) 4.2 mM. After 15 min incubation the sections were terminally fixed in 10% formal saline, washed in water, and counterstained with 1% methyl green. Finally, they were rinsed in acetone, dehydrated in alcohols, cleared in xylene and mounted in DPX (British Drug Houses Ltd, Poole, Dorset, UK).

Our estimation of the staining density of the various cells was qualitative. We used an arbitrary scale of 0 to + + +. The absence of any except minimal blue staining was recorded as 0, a clearly positive blue stain +, a strong blue + + and very strong blue verging on black + + +.

### Results

The depth of staining, assessed by eye on a scale 0 to + + +, is reported in Table I. There was no detectable difference between the five mice in the staining density of any particular tissue.

**Table I** Staining and grain counts

| *Tissue* | *NADPH* | *NADH* | *Grain count per 100μm² per exposure week* | *Ratio* |
|---|---|---|---|---|
| Liver | | | | |
| Periportal | 0 | + | 0.6 (0.06) | 4.0 (0.91) |
| Centrilobular | + + | + + + | 2.4 (0.12) | |
| Lung | | | | |
| Airway | + + + | + + + | 1.5 (0.04 ) | 6.7 (0.53) |
| Alveoli | + [a] | + [a] | 0.22 (0.038) | |
| Parotid gland | | | | |
| Duct | + + | + + | 0.184 (0.013) | 2.43 (0.13) |
| Parenchyma | 0 | 0 | 0.079 (0.004) | |
| Meibomian gland | + + | + + + | 13.0 (1.6 ) | 68 (14) |
| Stroma | 0 | 0 | 0.19 (0.03) | |
| Vulval gland | + + | + + | 4.17 (0.65) | 22 (3.8) |
| Sebaceous gland | + | + | 1.6 (0.2 ) | |
| Voluntary muscle | 0 | + | | |

The grain counts for liver, meibomian gland, stroma, lung (airway) and sebaceous gland have been previously published (Cobb & Nolan, 1989) and are from mice injected at 750 mg $kg^{-1}$ MISO (rel. spec. act. 74 MBq $mg^{-1}$). The remaining counts are from tissue prepared in the same study but not previously reported. The stroma was included to give data on background connective tissue levels. The standard error of the mean is in parentheses. The statistical comparisons of samples were by means of Student's *t* test. All ratios were significantly greater than 1 ($P<0.05$). [a] Positivity arose only from uniformly scattered cells, possibly type II pneumonocytes and/or macrophages. To calculate the grain count over the alveoli a measurement was first made of the ratio of alveolar area to alveolar wall and the grain count above alveolar wall was multiplied by this factor.

### *Liver*

The positive blue staining using either NADPH or NADH was largely restricted to the centrilobular zone throughout the liver. This is the same zone in which high MISO binding had previously been observed (Cobb & Nolan, 1989).

### *Meibomian (tarsal) gland*

This is a large, modified sebaceous gland which discharges near the base of the eyelashes. The whole gland was seen to stand out from the neighbouring tissue by virtue of its positive blue staining (Figure 1). Like the sebaceous and vulval glands the meibomian gland exhibits holocrine secretion. That is, the basal cells proliferate, then mature and hypertrophy, and the whole cell is finally voided through the duct. The basal cells of the acini were the most strongly positive. In the mature cells above the basal layer the blue staining was reduced and was at its lower level when the grossly enlarged and degenerating cells were at the point of extrusion into the main ducts. It was, however, predominantly in the ducts rather than the acini that the binding of MISO 24 h after injection had been observed. It should be remembered that the basal cells will in 24 h divide, mature and progress to the main ducts for secretion. In unpublished studies we have observed that 2 h after the injection of $^3$H-MISO the highest grain counts are over the basal cells, i.e. where the NBT stain was strongest in the present study. One other group of cells staining positively in the eyelid were the stratified epithelial cells of the conjuctival sac lining. Their retention of MISO was found to be variable.

**Figure 1** Meibomian gland. High NADPH tetrazolium reductase activity in the meibomian gland basal cells (g). The staining is less as the cells mature (arrows) and progress toward the main duct (d). The hair follicles of the eyelid (asterisk) are at the top of the figure and the stratified squamous lining of the conjunctival sac (c), which in parts stains positively, is to the left. The muscle of the eyelid (m) is negative, staining only with the counterstain methyl green. Bar represents 60 μm.

### Sebaceous gland

Again the formazan staining was at its heaviest in the basal cells and became diluted during maturation, as the cells progressed towards the hair follicle undergoing hypertrophy and nuclear disintegration on the way (Figure 2). On the other hand the grain count was highest over the disintegrating cells being voided into the hair follicle (Figure 3; previously published in Cobb *et al.*, 1989).

### Vulval gland

As with the previous two tissues the most densely stained cells were in the base of the acini and staining was reduced as the cells progressively expanded and degenerated prior to holocrine secretion. This was in contrast to the autoradiographic studies in which the most dense grain counts were observed above the degenerating cells in the ducts and not in the basal cells (Cobb *et al.*, 1989), although in fact the grain counts in the basal cells were above the surrounding stroma (Cobb & Nolan, 1989).

### Lung

Strong staining of the airway epithelium caused it to stand out clearly from all other tissues of the lung (Figures 4 and 5). The positive staining extended from the terminal airway up into the highest point of the trachea that was examined, i.e. distal third. There did not appear to be a difference in the staining between the different cell types of the epithelium (Figure 5) but the staining method might have been insufficiently sensitive for this degree of discrimination. The high reductase activity in the airways coincided with the raised grain count which was also observed only in the airways (Table I and Figure 6), extending from the terminal airways to the trachea. With neither histochemistry nor autoradiography was the technique sufficiently precise to be able to decide whether or not the thin mucociliary layer above the airway cells was involved.

**Figure 2** Sebaceous gland. High NADPH tetrazolium reductase activity is concentrated at the base of the paired sebaceous glands (arrow heads). The staining is less dense as the cells mature and progress towards the hair follicle. The keratinised stratified squamous epithelium of the skin surface shows some reductase activity, but the remaining skin components are negative. Bar represents 45 μm.

**Figure 3** Sebaceous glands (ARG). There is a high grain count over the mature cells (straight arrows). A reduced, but significantly above background, grain count is present above the basal cells (curved arrows). Stain, haematoxylin and eosin. Bar represents 45 μm.

**Figure 4** Airway epithelium. NADPH tetrazolium reductase activity outlining the airway epithelium (double and single arrows). By contrast the pulmonary veins (v) and arteries (a) are negative, identifiable only by the methyl green counterstain. Bar represents 210 μm.

### Parotid gland

The fomazan was observed only over the striated cells which line the intralobular ducts of the parotid gland (Figure 7). The striated appearance of these cells at light microscopy is

**Figure 5** Airway epithelium. NADH tetrazolium reductase activity in the terminal airway. The saw-tooth outline of the luminal surface is due to the protruding apices of Clara cells (arrow heads). Scattered throughout the alveolar walls are positive staining cells (arrows) which from their distribution could be type II pneumonocytes or, less likely, lung macrophages. Bar represents 75 μm.

**Figure 7** Parotid gland. NADH tetrazolium reductase activity in the lining epithelium of the ducts of the parotid salivary gland (arrows). The staining is heaviest in the basal half of the cells, which are densely packed with mitochondria. The cell nuclei appear as clear white circles on the luminal side of the darkly staining basal area. Bar represents 75 μm.

**Figure 6** Airway epithelium (ARG). The grains overlying the epithelium (between arrows) indicate the presence of bound $^3$H-MISO metabolite. The lumen of the airway is at the top of the figure. Stain, haematoxylin and eosin. Bar represents 20 μm.

due to densely packed mitochondria in the basal area. These were also the only parotid gland cells to exhibit a raised grain count in the autoradiographic studies (Table I).

*Voluntary muscle and stroma*

Positive staining voluntary muscle in the eyelids was restricted to approximately 1 in 6 fibres. These were the type I (slow) fibres which are rich in mitochondria and have previously been reported to be NBT reductase positive, using NADH (Bancroft, 1982). These fibres were not associated with bound MISO in our previous studies. The stroma in all tissues examined was negative for staining, as it had been for MISO binding.

### Discussion

The present results support our earlier suggestion that the binding of MISO to apparently well oxygenated tissues could in part be due to local high nitroreductase activity. Because the presumed normoxic, MISO-binding, cells were in small groups or layers it was not feasible to dissect them out for biochemical analysis. For this reason the histochemical approach has been used. The well established Hack and Helmy method for NADPH- and NADH-tetrazolium reductase indicates the presence of one or more dehydrogenases, including DT-diaphorase. It has previously been reported that DT-diaphorase is the only NAD(P)H dehydrogenase which is equally reduced by either of the two reduced pyridine nucleotides (Schor *et al.*, 1982). The similarity of staining using NADPH and NADH as cofactors in the present study therefore suggests the possibility of nitroreduction by DT-diaphorase in some of the tissues. However, the nitroblue tetrazolium stain is not specific and other reductases may well be involved, e.g. xanthine oxidase, aldehyde oxidase, cytochrome P-450 reductase and NADPH: cytochrome P-450 (cytochrome *c*) reductase. In their study of the complete ($6e^-$) nitroreduction of 2-nitroimidazole benznidazole by mouse liver Walton and Workman (1987) found that the microsomal enzymes NADPH: cytochrome P-450 (cytochrome *c*) reductase and cytochrome P-450 mainly were implicated. These enzymes are most active in the centrilobular zone of the liver (Van Noorden & Butcher, 1984).

The liver and the gastrointestinal tract are identified as the prime sites for nitroreduction (Chin & Rauth, 1981). It is therefore perhaps not surprising that MISO binding has been observed at high levels in the liver (Chin & Rauth, 1981; Maxwell *et al.*, 1989; Cobb *et al.*, 1990). On the other hand Van Os-Corby *et al.* (1987) were of the view that the liver has a sufficiently low $pO_2$ to produce MISO binding without the necessity for hypothesising the presence of high levels of nitroreductase. The relative importance of hepatic reductase capacity versus low hepatic $pO_2$ will not easily be resolved, particularly as the high reductase activity (and high MISO binding) we and others have observed is in the same site within the liver as the likely lowest $pO_2$, i.e. centrilobular zone (Pette & Brandau, 1966; Cobb & Nolan, 1989; Maxwell *et al.*, 1989). A number of workers have shown that hepatic nitroreductive enzymes are dependent on either NADPH or NADH (Gillette *et al.*, 1968; Poirier & Weisburger, 1974).

The sebaceous glands of the hair follicles and the modified sebaceous tissues, the meibomian and vulval glands, presented a similar histochemical picture, i.e. the highest staining in the basal cells of the acini with a tailing-off as the cells approached the ducts. This difference in distribution of activity is most probably explained by the constant maturation process in these glands. It would appear that maturation was associated with a loss in NBT reductase actitivty. The process of maturation might reasonably be expected to take about 24 h. Therefore, as labelled MISO became bound to the acinar cells shortly after injection it could be expected to be found at 24 h in the cells being extruded – which indeed is where high grain counts were observed. In unpublished work we have observed uniform grain counts over the acinar cells, including the basal cells, 2 h after the injection of $^3$H-MISO.

An incidental finding was positivity in the stratified epithelium of the skin and conjunctival sac. The subject of MISO retention in stratified epithelial cells (e.g. skin, oesophageal and stomach lining) has been raised elsewhere (Cobb *et al.*, 1989, 1990) and is a complex matter which is currently under study.

The combination of a high grain count, previously observed over the lung airway epithelium after the administration of $^3$H-MISO (Table I), and strong formazan staining, points to the possibility that local high reductase activity was overwhelming the ability of local oxygen to maintain futile cycling. An alternative possibility is a 2e$^-$ reduction involving enzymes such as DT-diaphorase effectively bypassing futile cycling and leading to the production of bound MISO (Mason, 1982; Iyanasi, 1987). We could not identify the reductase(s) involved although a similar staining pattern with both NADPH and NADH as substrates pointed to the possibility of at least some DT-diaphorase activity. Other enzymes should not be excluded, for example, NADPH cytochrome P-450 reductase, which has been observed in high levels in parts of the upper airways in rats (Reed *et al.*, 1986).

The positive staining of the cells lining the intralobular duct of the parotid gland mimicked closely the raised grain count observed in the autoradiographs in the previous studies. The $^3$H-MISO appeared to be bound to the striated cells, so-called because of the dense packing of mitochondria in the basal half of the cells. The blue stain was also densest in the basal half of the cells (Figure 7).

The case we are offering for a positive relationship between high local nitroblue tetrazolium reductase activity and MISO binding in the apparently normoxic tissues is by no means proven. Wherever we have previously observed bound MISO we have in the present histochemical study observed high NBT reductase activity; but the coincidence could be fortuitous. There might in fact be unsuspected hypoxia of many of these cells. While a possibility in the liver, this would seem unlikely in much of the airways.

The possibility should be considered that nitroreduction of MISO and related compounds to cytotoxic species might lead to cell damage in non-tumorous tissues which have high reductase capacity. While we have little evidence of this at present, there are two interesting papers by Knox *et al.* (1988*a*, *b*) which should be considered. In these they reported on the nitroreduction of CB1954 5-(aziridin-l-yl)-2, 4-dinitrobenzamide by DT-diaphorase in the rat. CB1954 is a cytotoxic drug, with radiosensitising properties, and a nitro group (Cobb *et al.*, 1969). Our interest in the papers by Knox *et al.* stems from the earlier toxicology of this drug in which histopathological changes were observed in the lung airway epithelium, the centrilobular areas of the liver, and the lens epithelium – a tissue lying within eight or so cells of the meibomian gland secretions (Cobb, 1970). The possibility exists that high DT-diaphorase levels in these tissues could have led to these histopathological changes following nitroreduction of CB 1954 to its cyctotoxic metabolite.

If MISO is reduced to the reactive metabolite in normoxic tissues by virtue of a high local reductase capacity, it would be of interest to examine tumours arising from such tissues for similarly high or possibly higher levels of reductase. Schor and colleagues have identified raised DT-diaphorase levels in hepatomas and Leydig cell tumours in the rat and mouse (Schor *et al.*, 1976, 1978; Schor & Morris, 1977) and in man, Koudstaal *et al.* (1975) and Watternburg (1959) have observed raised (above parent tissue) levels of NADH-tetrazolium reductase in colorectal and breast tumours. This approach is of current interest because of the development of bioreductive drugs for anti-tumour therapy (Stratford *et al.*, 1986; Adams & Stratford, 1988). It would seem that bioreductive drugs might fruitfully make headway in the area of high reductase tumours because of the possibility of activating pro-drugs in the normoxic as well as in the hypoxic cells.

We wish to thank Dr David Papworth for his statistical analyses and Dr Keith Morris for the calculation of alveolar wall area using the Seescan image-analyser.

### References

ADAMS, G.E. (1977). Hypoxic cell sensitizers for radiotherapy. In *Cancer: Comprehensive Treatise VI*, Becker, F.F. (ed.) p. 181. Plenum Press: New York.

ADAMS, G.E. & STRATFORD, I.J. (1988). Bioreductive radiation sensitizers. In *Progress in Radio-oncology IV*, Karcher, K.H. (ed.) p. 157. Proceedings of the Fourth Meeting on Progress in Radio-Oncology, Vienna. International Club for Radio-Oncology: Austria.

ASQUITH, J.C., WATTS, M.E., SMITHEN, C.E. & ADAMS, G.E. (1974). Electron affinic sensitization. V. Radiosensitization of hypoxic bacteria and mammalian cells *in vitro* by some nitroimidazoles and nitropyrazoles. *Radiat. Res.*, **60**, 108.

BANCROFT, J.D. (1982). Enzyme histochemistry. In *Theory and Practice of Histological Techniques*, 2nd edn, Bancroft, J.D. & Stevens, A. (eds) p. 398. Churchill Livingstone: Edinburgh.

CHAPMAN, J.D., RALEIGH, J.A., PEDERSEN, J.E. & 4 others (1979). Potentially three distinct roles for hypoxic cell sensitizers in the clinic. In *Proceedings of VIth International Congress of Radiation Research*, Okada, S., Imamura, M., Terashima, T. & Yamaguchi, H. (eds) p. 885. Japanese Association for Radiation Research: Tokyo.

CHIN, J.B., RAUTH, A.M. & VARGHESE, A.J. (1980). Pharmacokinetics and metabolism of misonidazole in C3H mice. In *Radiation Sensitizers: Their Use in the Clinical Management of Cancer*, Brady, L.W. (ed.) p. 474. Masson Publishing: New York.

CHIN, J.B. & RAUTH, A.M. (1981). The metabolism and pharmacokinetics of the hypoxic cell radiosensitizer and cytotoxic agent misonidazole, in C3H mice. *Radiat. Res.*, **86**, 341.

COBB, L.M. (1970). Toxicity of the selective antitumor agent 5-aziridino-2,4-dinitrobenzamide in the rat. *Toxicol. Appl. Pharmacol.*, **17**, 231.

COBB, L.M., CONNORS, T.A., ELSON, L.A. & 4 others (1969). 2, 4, dinitro-5-ethyleneimino-benzamide (CB1954): a potent and selective inhibitor of growth of the Walker carcinoma 256. *Biochem. Pharmocol.*, **18**, 1519.

COBB, L.M & NOLAN, J. (1989). Autoradiographic study of tritium labelled misonidazole in the mouse. *Int. J. Radiat. Oncol. Biol. Phys.*, **16**, 953.

COBB, L.M., NOLAN, J. & BUTLER, S.A. (1990). Tissue distribution of $^{14}$C- and $^3$H-labelled misonidazole in the tumour-bearing mouse. *Int. J. Radiat. Oncol. Biol. Phys.*, **18** (in the press).

COBB, L.M., NOLAN, J. & O'NEILL, P. (1989). Microscopic distribution of misonidazole in mouse tissues. *Br. J. Cancer*, **59**, 12.

FLOCKHART, I.R., LARGE, P., TROUP, D., MALCOLM, S.L. & MARTON, T.R. (1978). Pharmacokinetic & metabolic studies of the hypoxic cell radiosensitizer misonidazole. *Xenobiotica*, **8**, 97.

FRANKO, A.J. (1986). Misonidazole and other hypoxia markers: metabolism and applications. *Int. J. Radiat. Oncol. Biol. Phys.*, **12**, 1195.

FRANKO, A.J., RALEIGH, J.A., SUTHERLAND, R.G. & SODERLIND, K.J. (1989). Metabolic binding of misonidazole to mouse tissues. Comparison between labels on the ring and side chain, and the production of tritiated water. *Biochem. Pharmacol.*, **38**, 665.

GARRECHT, B.M. & CHAPMAN, J.D. (1983). The labelling of EMT-6 tumours in BALB/C mice with $^{14}$C-misonidazole. *Br. J. Radiol.*, **56**, 745.

GILLETTE, J.R., KAMM, J.J. & SESAME, H.A. (1968). Mechanism of p-nitro benzoate reduction in liver: the possible role of cytochrome P-450 in liver microsomes. *Mol. Pharmacol.*, **4**, 541.

HACK, M.H. & HELMY, F.M. (1964). *An Introduction to Comparative Correlative Histochemical Principles*. Gustav Fischer Verlag: New York.

HALL, E.J. & ROIZIN-TOWLE, L. (1975). Hypoxic sensitizers: radiobiological studies at the cellular level. *Radiology*, **117**, 453.

IYANASI, T. (1987). On the mechanisms of one- and two-electron transfer by flavin enzymes. *Chem. Scripta*, **27A**, 31.

KOUDSTAAL, J., MAKKINK, B. & OVERDIEP, S.H. (1975). Enzyme histochemical pattern in human tumours – II oxidoreductases in carcinoma of the colon and the breast. *Eur. J. Cancer*, **11**, 111.

KNOX, R.J., BOLAND, M.P., FRIEDLOS, F., COLES, B., SOUTHAN, C. & ROBERTS, J. (1988*a*). The nitroreductase enzyme in Walker cells that activates 5-(aziridin-1-yl)-2,4-dinitrobenzamide (CB1954) to 5-(aziridin-1-yl)-4-hydroxylamino-2-nitrobenzamide is a form of NAD (P) H dehydrogenase (quinone) (EC 1.6.99.2). *Biochem. Pharmacol.*, **37**, 467.

KNOX, R.J., FRIEDLOS, F., JARMAN, M. & ROBERTS, J. (1988*b*). A new cytoxic, DNA interstrand crosslinking agent, 5-(aziridin-1-yl)-4-hyroxylamino-2-nitrobenzamide, is formed from 5-(aziridin-1-yl)-2,4-dinitrobenzamide (CB1954) by a nitroreductase enzyme in Walker carcinoma cells. *Biochem. Pharmacol.*, **37**, 4661.

MASON, R.P. (1982). Free-radical intermediates in the metabolism of toxic chemicals. In *Free Radicals in Biology*, Pryor, W.A. (ed.) p. 161. Academic Press: New York.

MAXWELL, A.P., McMANUS, M.P. & GARDENER, T.A. (1989). Misonidazole binding in murine liver tissue: a marker of cellular hypoxia *in vivo*. *Gastroenterology*, **97**, 1300.

McMANUS, M.E., LANG, M.A., STUART, K. & STRONG, J. (1982). Activation of misonidazole by rat liver microsomes and purified NADPH-cytochrome and reductase. *Biochem. Pharmacol.*, **31**, 547.

MILLER, G.G., NGAN-LEE, J. & CHAPMAN, J.D. (1982). Intracellular localization of radioactivity labelled misonidazole in EMT-6 tumor cells *in vitro*. *Int. J. Radiat. Oncol. Biol. Phys.*, **8**, 741.

OLIVE, P.L. (1979). Inhibition of DNA synthesis by nitroheterocycles. II. Mechanisms of cytotoxicity. *Br. J. Cancer*, **40**, 94.

PETTE, D. & BRANDAU, H. (1966). Enzym-Histogramme, und Enzymaktivitatsmuster der Ratternleber. *Enzymol. Biol. Clin.*, **6**, 79.

POIRIER, L.A. & WEISBURGER, J.H. (1974). Enzyme reduction of carcinogenic aromatic nitro compounds by rat and mouse liver fractions. *Biochem. Pharmacol.*, **23**, 661.

RAUTH, A.M. (1984). Pharmacology and toxicology of sensitizers: mechanism studies. *Int. J. Radiat. Oncol. Biol. Phys.*, **10**, 1293.

REED, C.J., LOCK, E.A. & DEMATTEIS, F. (1986). NADPH: cytochrome P-450 reductase in olfactory epithelium. *Biochem. J.*, **240**, 585.

SCHOR, N.A. & CORNELISSE, C.J. (1983). Biochemical and quantitiative histochemical study of reduced pyridine nucleotide dehydrogenation by human colonic carcinomas. *Cancer Res.*, **43**, 4850.

SCHOR, N.A. & MORRIS, H.P. (1977). The activity of the DT-diaphorase in experimental hepatomas. *Cancer Biochem. Biophys.*, **2**, 5.

SCHOR, N.A., OGAWA, K., LEE, G. & FARBER, E. (1978). The use of DT-diaphorase for the detection of foci of early neoplastic transformation in rat liver. *Cancer Lett.*, **5**, 167.

SCHOR, N.A., RICE, B.F. & HUSEBY, R.A. (1976). Dehydrogenation of reduced pyridine nucleotides by Leydig cell tumors of the rat testis. *Proc. Soc. Exp. Biol. Med.*, **151**, 418.

SCHOR, N.A., STEDMAN, R.B., EPSTEIN, N. & SCHALLY, G. (1982). Rat splenic D-T diaphorase and NAD (P) H-nitroblue tetrazolium reductase. Their use to assess the action of polycyclic hydrocarbons in the lymphatic system. *Virchows Arch. Cell Biol.*, **41**, 83.

STRATFORD, I.J. & ADAMS, G.E. (1978). The toxicity of the radiosensitizer misonidazole towards hypoxic cells *in vitro*: a model for mouse and man. *Br. J. Radiol.*, **51**, 745.

STRATFORD, A.J., O'NEILL, P., SHELDON, P.W., SILVER, A.J.R., WALLING, J.M. & ADAMS, G.E. (1986). RSU 1069, a nitroimidazole containing an aziridine group: bioreduction greatly increases cytotoxicity under hypoxic conditions. *Biochem. Pharmacol.*, **35**, 105.

URTASUN, R.C., CHAPMAN, J.D., RALEIGH, J.A., FRANKO, A.J. & KOCH, C.J. (1986). Binding of $^{3}$H-misonidazole to solid human tumours as a measure of tumor hypoxia. *Int. J. Radiat. Oncol. Biol. Phys.*, **12**, 1263.

VAN NOORDEN, C.J.F. & BUTCHER, R.G. (1984). Histochemical localization of NADP-dependent dehydrogenese activity with 4 different tetrazolium salts. *J. Histochem. Cytochem.*, **32**, 998.

VAN OS-CORBY, D.J., KOCH, C.J. & CHAPMAN, J.D. (1987). Is misonidazole binding to mouse tissues a measure of cellular $pO_2$? *Biochem. Pharmacol.*, **36**, 3487.

VARGHESE, A.J., GULYAS, S.S. & MOHINDRA, J.K. (1976). Hypoxic-dependent reduction of 1-(-nitro-1-imidazoyl)-3-methoxy-2-propanol by Chinese hamster ovary cells and KHT tumor cells *in vitro* and *in vivo*. *Cancer Res.*, **36**, 3761.

VARGHESE, A.J. & WHITMORE, G.F. (1980). Binding to cellular macromolecules as a possible mechanism for the cytotoxicity of misonidazole. *Cancer Res.*, 40, 2165.

WALTON, M.I. & WORKMAN, P. (1987). Nitroimidazole bioreductive mechanism. Quantitation and characterization of mouse tissue – benznidazole nitroreductases *in vivo* and *in vitro*. *Biochem. Pharmacol.*, **36**, 887.

WATTERNBERG, L. (1959). A histochemical study of five oxidative enzymes in carcinoma of the large intestine in man. *Am. J. Pathol.*, **35**, 113.